# Association of dietary energy density with cardiometabolic risk factors and metabolic syndrome in Tehranian older adults

**DOI:** 10.34172/jcvtr.2020.17

**Published:** 2020-05-19

**Authors:** Hossein Shahinfar, Maryam Safabakhsh, Sara Mansouri, Kurosh Djafarian, Cain C. T. Clark, Sakineh Shab-Bidar

**Affiliations:** ^1^Department of Community Nutrition, School of Nutritional Sciences and Dietetics, Tehran University of Medical Sciences (TUMS), Tehran, Iran; ^2^Students’ Scientific Research Center (SSRC), Tehran University of Medical Sciences (TUMS), Tehran, Iran; ^3^Department of Clinical Nutrition, School of Nutritional Sciences and Dietetics, Tehran University of Medical Sciences, Tehran, Iran; ^4^Centre for Sport, Exercise, and Life Sciences, Coventry University, Coventry, CV15FB, UK

**Keywords:** Metabolic Syndrome, Obesity, Diet, Energy Intake

## Abstract

***Introduction:*** The objective of this study was to evaluate the association between the consumption of an energy-dense diet and cardiometabolic risk factors in Iranian older adults.

***Methods:*** This cross-sectional study was conducted on 226 older adults who were living in Tehran, Iran. Dietary energy density (DED) was calculated as energy per weight of food, kcal/g. The usual intake of participants was measured using a validated semi-quantitative food frequency questionnaire. Anthropometric measurements, fasting blood sugar, serum lipid profile and blood pressure and were assessed. The metabolic syndrome was defined according to National Cholesterol Education Program Adult Treatment Panel-III (NCEP ATP III).

***Results:*** Those who were in the third tertile of DED compared to the first tertile had 19% lower odds of having the cardiometabolic risk factors and metabolic syndrome (MetS) 0.81 (0.39,1.68) but the association was no significant (*P* =0.58). There was a significant inverse association between DED and systolic blood pressure (SBP) (β=-0.14, *P* =0.03) and diastolic blood pressure (DBP) (β=-0.17, *P* =0.01). We did not find any significant association between intake of energy-dense foods and serum levels of triglyceride (TG) (*P* =0.62), fasting blood sugar (FBS) (*P* =0.06), high-density lipoprotein (HDL) (*P* =0.72) and waist circumference (WC) (*P*=0.28).

***Conclusion:*** DED is negatively associated with SBP and DBP in Iranian older adults. Prospective studies are needed to establish a causal link between DED and MetS and risk factors of cardiovascular disease (CVD).

## Introduction

Metabolic syndrome (MetS) is characterized as a cluster of risk factors that elevates the chance of cardiovascular disease (CVD), type 2 diabetes (T2D)^1^ and all-cause of mortality.^2^ Various metabolic factors involved in the development of MetS, such as glucose intolerance (type 2 diabetes, impaired glucose tolerance, or impaired fasting plasma glucose), insulin resistance, abdominal obesity, dyslipidemia, and elevated blood pressure (BP).^3-5^ According to the National Cholesterol Education Program (NCEP) Adult Treatment Panel-III (ATP III) MetS has been as three or more of waist circumference (WC) >102 cm in males and WC >88 cm in females, fasting plasma glucose ≥110 mg/dL in both gender, or a known diagnosis diabetes, fasting serum triglyceride ≥150 mg/dL in both gender, fasting high-density lipoprotein (HDL) cholesterol <40 mg/dL in males and HDL<50 mg/dL in females, or BP ≥ 130/85 mm Hg in both gender.^6^

The MetS and its cardiometabolic risk factors in developed countries and worldwide are highly prevalent.^7,8^ A recent study in Iran has reported that the total prevalence of MetS is 32%.^9^ One of the most important factors that significantly leads to a high prevalence of the MetS is obesity and the major cause of obesity is positive energy balance.^10^ Some studies have reported positive associations between dietary energy density (DED) and energy intake.^11-13^ DED (kcal/g) is calculated as the amount of energy in a particular weight of food.^14^ Diets with high energy density include higher amount of refined grains, fat and added sugars while lower amount of whole grains, dietary fiber, vegetables and fruits.^15^ Adherence to such diets has been related to the risk of T2D.^16^ Results from some studies that have examined the relationships between DED and body-weight status are controversial.^13,17-20^ Pursuant to several researches no significant relationship between the intake of energy-dense diets and obesity have published^21,22^ or a significant association only in men.^23^ Other studies have presented a significant positive association between DED and obesity.^24-26^ To our knowledge, few studies presented the association of DED with MetS^11,18^ and no study has been conducted on older adults so far. Therefore, the objective of this study was to clarify the link between DED, cardiometabolic risk factors, and MetS in Tehranian elderlies.

## Subjects and Methods

### Study population

For our cross-sectional study which conducted between October 2015 and January 2016 on 226 older adults. We involved 65 men and 161 women with a mean age of 67.04 ± 5.77 years, called up to Tehran health centers. The mean of energy density equal to 0.75 reported by a previous study^27^ was used for calculating the sample size. A sample size of 248 was determined considering “α” = 0.05, “β” =0.1. Sampling of individuals was conducted using two-stage cluster sampling in 25 Tehran health centers. Participant’s inclusion criteria included enrolling older adults over 60 years’ old who abled to answer questions. Participants were excluded if they had type 1 diabetes, dialysis, or had clinical symptoms of any other disease like cancer and those with any disease that affect vitamin D metabolism. We also excluded those with reported energy intakes <500 and >3500 kcal/d.

### Dietary data and dietary energy density evaluation

To evaluate the habitual dietary intake a valid and reliable semi-quantitative food frequency questionnaire (SQ-FFQ) which contain 147-item applied.^28,29^ Educated nutritionists, asked the participants to report their intake frequency for each food item consumed during the past year in terms of the day, week, month and year. Then these reported intakes were converted to grams per day using standard existing guidelines also we summed each food’s energy to acquire total energy intake. For developing DED, we divided each daily energy intake of subject that reported (kcal/d) into the total weight of foods consumed (g/d). The available evidence is based on changes in weight of food intake so we did not consider the weight of drinks consumed.^13^

### Anthropometric measurements and blood pressure

Height of participants was measured without shoes by a wall stadiometer with precision close to of millimeter (Seca, Germany) and weight by electronic scale (Seca 808, Germany) near to 0.1 kg with thin clothes minus any heavy coat or raincoat. Body mass index (BMI) was computed by dividing kilogram of weight into the height’s square (m^ 2^). We determined waist circumference (WC) by a non-elastic tape fixed in the middle of the iliac crest and the lowest rib on the exhale. To assess BP, first, we demanded individuals to rest for at least 10 min. Blood pressure was then measured using a standard mercury sphygmomanometer, twice with a 5-minute interval, while participants were sitting. The mean of the two measurements was recorded as the participant’s BP.

### Laboratory parameters

Blood samples (10 mL) were collected among the hours of 7-10 am. from all of fasted subjects. Next, blood samples were gathered in acid-washed test tubes without anticoagulants. After keeping at ambient temperature for half-hour and initiation of clot, blood samples were centrifuged at 1500 g for 20 minutes. We held samples of serum at - 80°C till later experiments. Glucose was assayed by the enzymatic (glucose oxidase) colorimetric method.

Commercial kit (Pars Azmun, Tehran, Iran). Serum total cholesterol (TC) and high density lipoprotein-cholesterol (HDL-C) were measured using a cholesterol oxidase phenol amino antipyrine method, and triglyceride (TG) was measured using a glycerol-3 phosphate oxidase phenol amino antipyrine enzymatic method.

### Other measurements

Extra details about lifestyle was acquired via self-administered questionnaires included age, sex, educational level, smoking, medical history and current use of medications. Smoking status was divided into current, former or never smoking. Education level was stated as the greatest degree of school reached and subjects were classified into either primary school or less, technical-professional school, secondary school, university or higher.

### Physical activity level

A validated short form of the International Physical Activity Questionnaire (IPAQ)^30^ was used to assess the level of physical activity for each participant and then classified into three level ; i.e. vigorous, moderate and light physical activity according to calculated Metabolic Equivalents (METs) over the past week very low (<600 MET-minutes/week), low (600-3000 MET-minutes/week), and moderate and high (>3000 MET-minutes/week).^31^

### Statistical methods

All statistical analyses were performed using the Statistical Package for the Social Sciences (SPSS version 25; SPSS Inc.) We considered *P* < 0.05 as significance level. DED was classified into low‐ (T1: <1.055 kcal/g), medium‐ (T2: 1.056-1.25 kcal/g), and high‐ (T3: >1.26 kcal/g) density tertiles. One-way analysis of variance (ANOVA) for continuous variables and chi-square analysis for categorical variables was used to compare general characteristics and dietary intake across tertile of DED. Food and nutrient intake were controlled for age and energy to carry out analysis of covariance (ANCOVA) among DED tertiles. Various models presented to discover the relationship between DED and MetS and cardiometabolic risk factors by means of multivariable logistic regression analysis. At first, we controlled for the confounding impact of age and sex. In the next model, more adjustments for smoking, physical activity, socio-economic status and BMI were accomplished. The overall trend of odds ratios across tertiles of DED was calculated by considering the median of DED in each tertile as a continuous variable.

## Results

General characteristics of study participants by tertiles of DED are presented in Table 1. In comparison with those in the first tertile, subjects in the last tertile were younger. There were no significant differences in other characteristics across tertiles of DED.

**Table 1:** General characteristics of study participants by tertiles of dietary energy density

	**All** **Mean ±SD**	**Tertiles of DED**			***P*** **value**
		**T1** **<1.055 kcal/g**	**T2** **1.056-1.25 kcal/g**	**T3** **>1.26 kcal/g**	
n	226	75	76	75	
Age (y)	67.04±5.77	67.78± 6.29	67.02±5.72	66.32±5.23	0.29
Weight (kg)	72.62±12.04	72.79±12.50	72.64±11.82	72.42±11.95	0.98
WC (cm)	99.22±10.37	100.04±10.70	99.80±10.48	97.82± 9.93	0.35
BMI (kg/m^2^)	29.75±4.53	30.30±5.13	29.18±4.09	29.79±4.32	0.31
Sex, n (%)					0.68
Male	65 (28.8)	29 (12.8)	18 (8.0)	18 (8.0)	
Female	161 (71.2)	46 (20.4)	58 (25.7)	57 (25.2)	
Marital status, n (%)					0.59
Single	3 (1.3)	1 (0.4)	2 (0.9)	0 (0.0)	
Married	161 (71.2)	51 (22.6)	55 (24.3)	55 (24.3)	
Divorced	4 (1.8)	2 (0.9)	2 (0.9)	0 (0.0)	
Widow	58 (25.7)	21 (9.3)	17 (7.5)	20 (8.8)	
Smoking, n (%)					0.97
Non-smoker	191 (84.5)	64 (28.3)	64 (28.3)	63 (27.9)	
Former and current smoker	39 (15.5)	11 (4.9)	12 (5.3)	12 (5.3)	
Physical^*^ activity, n (%)					0.33
Very low	108 (47.8)	38 (16.8)	39 (17.3)	31 (13.7)	
Low	78 (34.5)	22 (9.7)	23 (10.2)	33 (14.6)	
Medium and high	40 (17.7)	15 (6.6)	14 (6.2)	11 (4.9)	

*P* value less than 0.05 was considered significant. Values are based on mean ± standard deviation or reported frequency (percentage). One-way ANOVA for quantitative data and Chi-2 test for qualitative data have been used. DED: dietary energy density; BMI: body mass index; WC: waist circumference. *Defined using METs were classified as very low (< 600 MET-minutes/week), low (600-3000 MET-minutes/week), and moderate and high (> 3000 MET-minutes/week).

Dietary intake of macronutrients and food groups according to the tertiles of the DED were indicated in Table 2. Significant differences were demonstrated in dietary intake of carbohydrate (*P*  < 0.001), protein (*P*  < 0.001), total fat (*P*  < 0.001), cholesterol (*P*  = 0.007) and fiber (*P*  < 0.001) across the tertiles of DED. Subjects in the third tertile of DED had higher intake of carbohydrate, protein, total fat, cholesterol and fiber compared to the first tertile. Significant differences were also observed in terms of vegetables (*P =* 0.02), refined grains (*P*  < 0.001), nuts and legumes (*P*  < 0.001) among the tertiles of DED. Individuals in the last tertile of DED had higher intake of fruits, meat and fish, refined grains and nuts and legumes as well as had lower intake of vegetables. The intake of whole grains and dairy was not significantly different across tertiles of DED.

**Table 2:** Dietary intake of nutrients/foods according to the tertiles of the dietary energy density

	**All** **Mean ±SD**	**Tertiles of DED**			***P*** value*	***P*** value**
		**T1** **<1.055 kcal/g**	**T2** **1.056-1.25 kcal/g**	**T3** **>1.26 kcal/g**		
n	226	75	76	75		
Energy, kcal/d	2775±799	2749±911	2731±68	2846±793	0.63	0.45
Carbohydrates, g/d	371.2±137.2	300.7±87.8	361.5±102.2	451.4±165.1	<0.001	<0.001
Added sugar, g/d	151.4±62.4	125.0±39.5	148.1±47.6	181.1±79.7	<0.001	<0.001
Protein, g/d	84.1±31.7	70.3±20.2	81.2±23.7	100.8±39.9	<0.001	<0.001
Total fat, g/d	78.9±36.4	59.0±17.3	77.6±21.4	100.1±49.2	<0.001	<0.001
Saturated fat, g/d	23.8±10.1	19.3±6.2	23.7±7.3	28.3±13.4	<0.001	<0.001
Cholesterol, mg/d	204.8±103.4	177.2±66.1	206.8±99.7	230.3±129.0	0.007	0.002
Fiber, g/d	46.7±24.7	36.1±11.9	43.5±18.5	60.6±32.3	<0.001	<0.001
Fruits, g/d	519.2±291.9	501.3±261.3	509.8±266.2	546.3±343.1	0.60	0.34
Vegetables, g/d	421.7±21.6	473.1±273.1	375.7±163.6	417±196.7	0.02	0.11
Meat and fish, g/d	55.8±39.7	49.8±27.6	53.5±33.8	64.1±52.6	0.07	0.02
Whole grains, g/d	20.0±32.6	21.2±42.9	16.9±20.9	22.0±30.5	0.58	0.88
Refined grains, g/d	454.9±220.4	329.2±143.5	445.4±160.1	590.0±258.0	<0.001	<0.001
Dairy, g/d	385.3±229.5	385±200	420.6±235.7	349.8±246.9	0.16	0.34
Nuts and legumes, g/d	48.4±38.9	37.1±21.3	44.9±28.19	63.3±54.5	<0.001	<0.001

DED: dietary energy density. *P* value less than 0.05 was considered significant. Values are based on mean ± standard deviation. *One-way anova, **Values adjusted for age and energy intake. Data for energy intake have just been adjusted for age.

The Multivariate adjusted means for TG, systolic BP (SBP), diastolic BP (DBP), fasting blood sugar (FBS), HDL-C and WC according to the tertiles of DED is shown in Table 3. In the crude model, we observed that higher DED was contributed to the lower DBP (*P =* 0.007) and SBP (*P =* 0.02) and there was no significant difference in terms of other components of MetS across tertiles of DED. After controlling for covariates these associations were remained non-significant except for DBP (*P =* 0.02).

**Table 3:** The Multivariate adjusted means for metabolic syndrome’s components according to ter-tiles of dietary energy density

	**All** **Mean ±SD**	Tertiles of DED*			***P*** **value**	***P*** value**
		**T1** **<1.055 kcal/g**	**T2** **1.056-1.25 kcal/g**	**T3** **>1.26 kcal/g**		
TG^£§^(mg/dL)	176.3±88.1	179.5±102.2	175.0±71.2	174.5±89.5	0.73	0.93
SBP^£§^ (mm Hg)	142.0±22.0	146.8±25.6	140.5±19.2	138.8±20.1	0.02	0.07
DBP^£§^(mm Hg)	84.6±14.7	88.7±18.6	84.1±12.1	81.7±11.8	0.007	0.02
FBS^£§^(mg/dL)	110.4±41.6	106.5±36.4	107.0±44.7	117.8±42.8	0.10	0.25
HDL-C^£§^(mg/dL)	49.1±12.9	47.4±13.9	50.8±12.2	49.0±12.5	0.47	0.46
WC (cm)	99.2±10.3	100.0±10.7	99.8±10.4	97.8±9.9	0.19	0.35
BMI (kg/m^2^)	29.7±4.5	30.3±5.1	29.2±4.1	29.8±4.3	0.31	0.31

DED: dietary energy density,TG: triglyceride, SBP: systolic blood pressure, DBP: diastolic blood pressure, FBS: fasting blood sugar, HDL-C: high density lipoprotein-cholesterol, WC: waist circum-ference, BMI: body mass index Data are means ± SD. * Dietary energy density was based on food only, no beverages. **All values adjusted for age, sex, cigarette smoking, physical activity, socioeconomic status. § Also adjusted for BMI. £Also adjusted for WC.

Multivariate adjusted odds ratios and 95% confidence intervals for MetS and its components across tertiles of DED are presented in Table 4. In crude model, although those who were in the third tertile of DED compared to the first tertile were less likely to have cardiometabolic risk factors and MetS [OR= 0.81; CI95%: 0.39,1.68], there was no association between higher DED and MetS (*P =* 0.58). After adjusting for age and sex also this result remained non-significant. There was also no significant association between DED and components of the cardiometabolic risk factors and MetS even after controlling for covariates.

**Table 4:** Multivariate adjusted odds ratios and 95% confidence intervals for metabolic syn-drome and its components across tertiles of dietary energy density

	**Tertiles of DED**			P value
	**T1** **<1.055 kcal/g**	**T2** **1.056-1.25 kcal/g**	**T3** **>1.26 kcal/g**	
Metabolic Syndrome				
Crude	1.00	1.20 (0.56, 2.56)	0.81 (0.39, 1.68)	0.58
Model 1^*^	1.00	1.20 (0.56, 2.56)	0.81 (0.39, 1.70)	0.59
Model 2^§^	1.00	1.01 (0.46, 2.24)	0.68 (0.31, 1.46)	0.50
Hypertriglyceridemia				
Crude	1.00	1.20 (0.63, 2.28)	0.85 (0.44, 1.61)	0.57
Model 1	1.00	1.21 (0.64, 2.32)	0.85 (0.44, 1.62)	0.54
Model 2	1.00	1.15 (0.59, 2.25)	0.76 (0.39, 1.50)	0.47
Hypertension				
Crude	1.00	0.92 (0.44, 1.97)	0.77 (0.38, 1.56)	0.82
Model 1	1.00	0.79 (0.38, 1.65)	0.76 (0.38, 1.54)	0.83
Model 2	1.00	1.00 (0.95, 1.07)	0.62 (0.29, 1.30)	0.58
Hyperglycemia				
Crude	1.00	1.13 (0.58, 2.21)	1.49 (0.76, 2.92)	0.47
Model 1	1.00	1.15 (0.58, 2.25)	1.53 (0.78, 3.01)	0.44
Model 2	1.00	1.17 (0.58, 2.37)	1.70 (0.83, 3.45)	0.31
Low HDL-C				
Crude	1.00	1.21 (0.63, 2.33)	0.74 (0.38, 1.46)	0.35
Model 1	1.00	1.20 (0.62, 2.31)	0.68 (0.34, 1.35)	0.25
Model 2	1.00	1.05 (0.52, 2.10)	0.56 (0.27, 1.18)	0.18
Abdominal obesity				
Crude	1.00	1.16 (0.56, 2.40)	0.87 (0.43, 1.77)	0.74
Model 1	1.00	1.14 (0.55, 2.36)	0.87 (0.42, 1.79)	0.77
Model 2	1.00	0.62 (0.25, 1.54)	0.48 (0.19, 1.21)	0.29

HDL-C: high density lipoprotein-cholesterol. Data are OR (95% CI). *Adjusted for age and sex. § Adjusted for age, sex, smoking, physical activity, socioeconomic status, body mass index and energy.

The associations between DED and components of MetS are shown in Table 5. Among all the components of MetS, SBP (β=-0.14, *P =* 0.02) and DBP (β=-0.16, *P =* 0.01) had a significant inverse association with DED. Moreover, after controlling for covariates these associations remained significant for SBP (β=-0.14, *P =* 0.03) and DBP (β=-0.17, *P =* 0.01). There was no significant association between intake of ED foods and serum levels of TG (*P =* 0.62), FBS (*P =* 0.06), HDL (*P =* 0.72) and WC (*P =* 0.28).

**Table 5:** Association between components of metabolic syndrome and dietary energy density

	**DED**			
	**β±SE**	**95% CI**	**R** ^ 2 ^	**P value**
TG (mg/dL)				
Model 1	-0.04±0.0001	0.0001,0.0001	0.002	0.53
Model 2	-0.03±0.0001	0.0001,0.0001	0.02	0.62
SBP (mm Hg)				
Model 1	-0.14±0.001	-0.003,0.0001	0.02	0.02
Model 2	-0.14±0.001	-0.003,0.0001	0.04	0.03
DBP (mm Hg)				
Model 1	-0.16±0.001	-0.005,-0.001	0.02	0.01
Model 2	-0.17±0.001	-0.005,-0.001	0.05	0.01
FBS (mg/dL)				
Model 1	0.12±0.0001	0.0001,0.002	0.01	0.07
Model 2	0.13±0.0001	0.0001,0.002	0.04	0.06
HDL-C (mg/dL)				
Model 1	0.008±0.001	-0.002,0.003	0.0001	0.90
Model 2	-0.02±0.001	-0.003,0.002	0.02	0.72
WC (cm)				
Model 1	-0.07±0.002	-0.005,0.002	0.005	029
Model 2	-0.07±0.002	-0.005,0.002	0.02	0.28

β: standardized coefficients, SE: standard error, CI: confidence interval, R2: R square, DED: dietary energy density, TG: triglyceride, SBP: systolic blood pressure, DBP: diastolic blood pressure, FBS: fasting blood sugar, HDL-C: high density lipoprotein-cholesterol, WC: waist circumference. *P* value less than 0.05 was considered significant. *P* value obtained from Linear regression. Model 1: Crude. Model 2: Adjusted for age, sex, smoking, socioeconomic status, physical activity and energy.

## Discussion

In the current cross-sectional study, we found a significant inverse association between DED with SBP and DBP. However, our findings showed a negative relationship but not significant between DED and odds of having MetS and other cardiometabolic risk factors in spite of controlling for familiar potential confounders. As far as we are know, this is the first study that investigates the association between DED and the MetS in older adults in the Middle East based on dietary data from validated FFQ.

In this research, we showed that higher intake of energy-dense foods were significantly related to decreased both SBP and DBP. It should be noted that no study has reported such a relation between DED and SBP and DBP. Although some studies failed to show any significant relationship between higher consumption of ED foods, SBP and DBP,^12,17,32^ the Swedish Obese Subjects Study (SOS) study indicated a positive association of adherence to ED pattern with SBP and DBP.^33^ This could be due to that ED diets are rich in fat, with higher level of energy production per gram, in compared with poor in vegetable, fruit, and fiber.^34-36^ A recent cross-sectional study suggested that higher consumption of whole-grain may improve cardiovascular function.^37^ Also, one current systematic review and meta-analysis demonstrated that high intake of fruits and vegetables decreases CVD risk.^38^ As well as some other meta-analyses indicate that consumption of nuts and legumes have beneficial effects on CVD risk.^39,40^ However, in an epidemiologic research, high processed and red meat consumption were related to elevated BMI and SBP.^41^

Our findings also suggest no significant association between DED and WC. In agreement with our findings a current cross-sectional study failed to report any significant relationship between DED and WC in Japanese men.^42^ Results of a prospective study al so showed that there is not any significant relationship between DED and WC after controlling for weight change.^20^ Moreover, a recent systematic review and meta-analysis of observational studies offered that DED is related to risk of increased adiposity, greater body weight change, but not BMI and WC.^34^ Conversely, Mendoza et al found a significant positive association between consumption of ED foods and WC.^18^ This result is similar to those reported in some other previous studies.^43,44^

According to our results, no significant association of DED with FBS and lipid profile was indicated. In line with our result, previous studies failed to show any significant relationship between DED and FBS.^11,32^ Murakami et al similarly did not find any significant link between DED and serum HDL and TG. In contrast, in a longitudinal study among Iranian adults DED was inversely associated with HDL-C and positively related to atherogenic index of plasma changes.^45^ In another epidemiologic research in Iran, findings showed that higher intake of ED foods lead to higher serum HDL and TG in female nurses.^12^

The discrepancies among studies may be partly related to specific characteristics of diet in each population because DED do not differentiate foods in terms of energy. In populations with greater adherence to Mediterranean diet with higher content of fat, the beneficial effects of this patterns are related to olive oil consumption.

The other reason for conflicting results may be due to the method of dietary assessment in studies. Some studies like the study conducted by Mendoza et al, used 1-day 24-hour dietary recall in their study.^18^ In contrast, we used a validated FFQ for dietary assessment which reflects individual’s long-term habitual dietary intakes.^48^ Additionally, in a study by Murakami et al, the whole picture of the MetS did not considered^17^ which may explain the findings of non-significant association between DED and metabolic risk factors among young Japanese women.^17^

In our research, mean and standard deviation of DED was 1.18 ± 0.25 kcal/g. This result was noticeably lower than that showed in eastern and western studies (1.79-1.85 kcal/g),^15,17,18^ which is related to the age of participants. Needs of energy among older adults are lower than in other age groups. Also, this could be because of higher intake of rice and traditional bread in eastern countries and higher consumption of fats and sugar in western countries ^13,15^ though the content of sugar and fat was the reason of high density of energy in their population.

Measurement of DED is still controversial. Many different methods are used to measure the energy density of foods, which their main difference is considering the contribution of drinks.^49,50^ A systematic review concluded that it is better to calculate DED based on foods, not the total amount of food and drink because considering drinks makes bias toward significant relationship.^51^ Many studies have shown that the impact of DED on weight and obesity is according to energy density of foods not drinks^10,52-54^ and reducing DED is an important aspect of prevention of gain weight and obesity. We found that among macronutrients, fat intake was positively associated with DED. It has been shown that due to higher density of fat in energy production than protein and carbohydrate, diets with higher content of fat tend to be energy dense.^55^ To be noted that fat content varies substantially in individual foods. Epidemiological evidence indicates that high-fat/low-fiber diets promote the chance of developing diabetes by 89% as compared to the low-fat/high-fiber diets.^17^

Several physiologic mechanisms have been suggested for the association of DED and MetS. Insulin resistance was determined to play a key role in the pathogenesis of the MetS^15^ and accumulating evidence suggests that intake of energy-dense foods may change insulin resistance, independent of obesity.^46^ First, higher DED is positively linked to higher content of refined grains and added sugars.^56^ Higher content of sugar in diet is also associated with higher glycemic load, which may further insulin resistance and increases the MetS and T2D risk.^56^ Second, in line with other studies, we also found that the content of total fat and saturated fat was higher in the top tertile as compared to the first tertile.^56^ According to the previous studies higher total fats and saturated fatty acids are positively linked with insulin resistance.^57^ Third, previously it has been presented that foods with greater DED are related to excess energy intake which lead to overweight and obesity.^13,57^

Variety of dietary assessment methods used, different populations in studies, and number and type of variables used as confounding factors may explain inconsistency among results. Moreover, the method of DED calculation based on using or not using beverages in the calculation, may result in different associations, especially because beverages may weaken associations with outcome measures may result in an increase within-person variation.^58^

Our study has also some limitations. First, the cross-sectional nature of the study precludes us from inferring causal associations. Longitudinal studies are needed to check causal relationships. We used semi-quantitative FFQ for gathering dietary data in spite of long term nutritional assessment of subjects but some foods that did not exist in FFQ have been not covered in calculation of DED.

## Conclusion

In summary, findings of this research indicated that higher DED is inversely related to SBP and DBP. Findings also suggest that higher intake of energy dense foods was not associated with risk of MetS and cardiometabolic risk factors. Further well-designed studies are required to investigate the causal relationship between DED and MetS.

## Competing interests

None.

## Ethical approval

All procedures were in accord with the ethical standards of the Tehran University of Medical Sciences (ethic Number: IR.TUMS.REC.1395.2618), who approved the protocol and informed consent form. All participants signed a written informed consent prior to the start of the study.

## Funding

This manuscript has been granted by Tehran University of Medical Sciences (Grant No: 27810).

## Acknowledgments

We thank the health centers for their collaboration. We sincerely appreciate all the subjects for their participation in this study. SS-b designed and supervised the study. SM helped intellectually in finalizing the study design. HS and MS analyzed data and wrote the preliminary manuscript and. All statistical analyses were done under the supervision of SSb. KD, SSb and CC critically revised the manuscript. All authors read and approved the final manuscript.
